# Direct Processing and Storage of Cell-Free Plasma Using Dried Plasma Spot Cards

**DOI:** 10.1021/acsmeasuresciau.2c00034

**Published:** 2022-07-25

**Authors:** Keith R. Baillargeon, Giorgio Gianini Morbioli, Jessica C. Brooks, Philip R. Miljanic, Charles R. Mace

**Affiliations:** Department of Chemistry, Laboratory for Living Devices, Tufts University, Medford, Massachusetts 02155, United States

**Keywords:** paper microfluidics, paper analytical devices, μPAD, dried plasma spot cards, point-of-care, bioanalysis, hematology

## Abstract

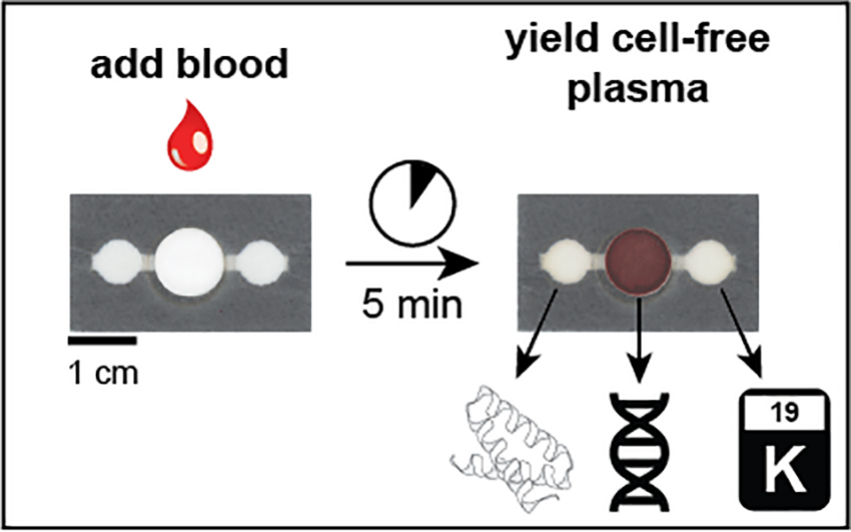

Plasma separation cards represent a viable approach for expanding testing capabilities away from clinical settings by generating cell-free plasma with minimal user intervention. These devices typically comprise a basic structure of the plasma separation membrane, unconstrained porous collection pad, and utilize either (i) lateral or (ii) vertical fluidic pathways for separating plasma. Unfortunately, these configurations are highly susceptible to (i) inconsistent sampling volume due to differences in the patient hematocrit or (ii) severe contamination due to leakage of red blood cells or release of hemoglobin (i.e., hemolysis). Herein, we combine the enhanced sampling of our previously reported patterned dried blood spot cards with an assembly of porous separation materials to produce a patterned dried plasma spot card for direct processing and storage of cell-free plasma. Linking both vertical separation and lateral distribution of plasma yields discrete plasma collection zones that are spatially protected from potential contamination due to hemolysis and an inlet zone enriched with blood cells for additional testing. We evaluate the versatility of this card by quantitation of three classes of analytes and techniques including (i) the soluble transferrin receptor by enzyme-linked immunosorbent assay, (ii) potassium by inductively coupled plasma atomic emission spectroscopy, and (iii) 18S rRNA by reverse transcriptase quantitative polymerase chain reaction. We achieve quantitative recovery of each class of analyte with no statistically significant difference between dried and liquid reference samples. We anticipate that this sampling approach can be applied broadly to improve access to critical blood testing in resource-limited settings or at the point-of-care.

## Introduction

Plasma—the liquid portion of whole blood—contains nearly 90% water to carry cells (e.g., red blood cells, RBCs; white blood cells, WBCs) and dissolved solutes (e.g., proteins, ions, and small molecules) throughout the body.^1,2^ The majority of these solutes by mass are proteins, such as albumin and immunoglobulins, with the remaining portion comprising nucleic acids, dissolved gases, ions, nutrients, and waste products from various tissues.^3^ The diversity of biomolecules in circulating plasma renders it a powerful biological sample for the evaluation of a patient’s health status. However, many diagnostically relevant analytes exist in trace quantities in this very complex sample matrix, which routinely requires additional processing (e.g., plasma separation from whole blood) and isolation (e.g., nucleic acid extraction) steps to be conducted prior to testing to minimize assay interferences and improve limits of detection.^4,5^ Separation of plasma from the cellular component of whole blood is routinely achieved by centrifugation in the laboratory or clinic where whole blood samples are submitted for testing. Separated plasma may be stored at ≤ −20 °C until analysis to maintain analyte stability.^6^ Outside of these centralized settings, plasma separation and storage face challenges related to the lack of electricity, clinical analyzers, and reliable cold-chain storage.^7,8^

In response, myriad technologies have been developed to separate plasma at the point-of-care, each requiring different degrees of user input. The least labor-intensive method is passive separation (e.g., hydrodynamic flow,^9^ acoustophoresis,^10^ sedimentation,^11^ agglutination,^12^ and filtration^13^), where the user only needs to add the sample to perform separation. In contrast, the most labor-intensive method is active separation (e.g., eggbeater,^14^ salad spinner,^15^ paperfuge,^16^ and fidget spinner^17^), where the user manually performs an action to separate plasma. Each approach presents certain advantages and limitations depending on the available resources and downstream applications. For example, passively storing dried blood or plasma in a hydrophilic porous matrix enhances the stability of analytes without the need of refrigeration or cold chain storage (e.g., up to 14 days at 25 °C).^18−20^ Similarly, drying samples of whole blood on a hydrophobic substrate—in the form of two-dimensional spheroids—can extend the stability of labile analytes for more than 30 days; however, this approach has yet to be integrated with plasma separation techniques.^21^ In contrast, active plasma separation technologies produce liquid plasma for immediate use with a diagnostic test, which obviates the need for cold-chain storage. However, if blood samples are collected in a remote setting without access to immediate testing or refrigeration, then analyte stability and diagnostic utility of the plasma will be compromised.^22^

Plasma separation cards (PSCs) utilize passive filtration and represent an excellent option for the processing and storage of plasma samples at the point-of-care due to the associated ease of collection, low cost, and ability to ship through the mail for off-site analysis.^23^ First generation PSCs utilized either vertical^24^ or lateral^25,26^ flow plasma separation membranes (PSM), often paired with a porous cardstock (e.g., Whatman CF12, Ahlstrom 226, and Munktell TFN) to produce cell-free plasma. In these formats, the distribution of plasma collected by the porous material is both unrestricted and undefined, which presents two distinct challenges: (i) variable sample output volume and (ii) chromatographic effects. The first is directly related to the hematocrit—the ratio of packed RBCs to total volume of plasma—which ranges from 36–50% (normal values),^27^ representing a highly variable liquid plasma volume from a fixed volume of whole blood. For example, unrestricted flow of plasma produced from 75 μL of blood could result in a spot containing as much as 48 μL or as little as 37.5 μL of plasma, depending on the hematocrit value (not including the loss of volume to the materials). Additionally, unrestricted flow of plasma over an undefined collection area may result in a concentration gradient of analytes, which is dependent on the specific sampling location along the material (i.e., subpunch), as demonstrated by lateral flow devices.^28^ For example, the spiral shape of the HemaSpot-SE device utilizes lateral separation and yields a mixture of cells (RBCs and WBCs) at the center while gradually transitioning to cell-free plasma toward the “tail-end” of the spiral membrane.^26^ Utilizing only lateral flow to perform separation does not generate replicate samples due to the chromatographic effect along the spiral shape. Inconsistent sampling of plasma due to hematocrit-dependent bias and chromatographic effects presents two major limitations for quantitative analysis using current PSCs.

Over the past decade, several approaches have been developed to specifically address the challenges related to quantitative sampling using PSCs (e.g., inconsistent/low sample output volumes). The resulting devices include unique features such as a singular collection tab (Cobas PSC, Roche Diagnostics),^29^ two plasma collection discs from a single input of whole blood (DUO, Novilytic),^30^ and water-soluble films (“volume-defined DPS”).^31^ The Cobas PSC can process a larger volume of sample (140 μL input volume of blood)^32^ and stores the plasma in a defined shield-shaped zone to increase the speed of wicking into the porous material. While this card produces a larger volume of dried plasma (ca. 40 μL), it is specifically designed for integration with associated Roche clinical analyzers, which further limits the use of a single card to a single assay or platform.^33^ The Novilytic DUO card includes a spreading layer beneath the PSM to distribute cell-free plasma to two precut paper discs; however, the maximum output volume is only 3.8 μL per disc and may preclude quantitation of analytes in low abundance (e.g., hormones, cytokines, and circulating nucleic acids). Both the Novilytic DUO cards and Cobas PSCs utilize only vertical separation to store plasma directly beneath the separation material, which can result in contamination due to hemolysis (from shearing or upon drying) or leakage of RBCs through the PSM.^34^ Alternatively, the “volume-defined DPS card” provides consistent metering of plasma utilizing lateral flow channels and overflow control for samples with a low hematocrit by inclusion of a water-soluble poly(vinyl alcohol) film. This device functions well as a stand-alone plasma separator unit but requires a complete seal for separation, has an open structure, and may not be amenable to multiplexing to produce multiple unique samples of plasma. Utilizing lateral flow separation membranes or singular collection zone approaches would require a clinician to collect multiple cards—potentially with multiple fingersticks—to perform immunoassays (to detect proteins) and perform PCR (to detect nucleic acids). Multiple blood collections can potentially cause increased pain and discomfort for the patient and increase sampling errors during the analytical process due to hemolysis. Increasing the number of tests possible from a single collection card could address these challenges and expand access to critical health information.

Recently, we reported a patterned dried blood spot card (pDBS) for improved sampling of whole blood from fingerstick volumes of blood.^35^ Our pDBS card utilized hydrophobic wax barriers to control the application, distribution, and storage of whole blood into four distinct sample collection zones. Restricting the flow of whole blood with wax barriers allowed enhanced sampling, and a consistent output volume contained in each paper disc, independent of the hematocrit. Our initial investigation into the development of the pDBS card included an evaluation of three paper types and partial sealing with laminate sheets to prevent uneven evaporation. We separately reported an assembly of porous materials for the separation of plasma from whole blood, yielding threefold separation efficiency using only passive filtration.^36^ By integrating these two approaches, we aimed to provide on-chip separation of the cellular (i.e., RBCs, WBCs, and platelets) and liquid (i.e., plasma) components of whole blood at the point-of-care while maintaining enhanced sampling for accurate quantitative analysis in centralized laboratories.

Herein, we present a patterned dried plasma spot (pDPS) card capable of passively producing two discrete, metered volumes of plasma from a single input of whole blood that is independent of the hematocrit. Spatially separating the collection zones away from the separation materials yields high-quality, cell-free plasma samples with no detectable hemolysis. Additionally, we utilize the enriched, dried blood spot from the blood inlet zone to yield a third sample to analyze cellular DNA. We broadly demonstrate the versatility of this card by quantification of representative classes of analytes and techniques including (i) the soluble transferrin receptor by enzyme-linked immunosorbent assay (ELISA), (ii) potassium by inductively coupled plasma atomic emission spectroscopy (ICP-AES), and (iii) 18S rRNA by reverse transcriptase quantitative polymerase chain reaction (RT-qPCR). Preparing different analytes from a single card can permit more tests to be performed without requiring multiple blood collections. This work is a step forward toward the adoption of more effective point-of-care tools for serological and nucleic acid testing.

## Experimental Design

### Card Design

**Figure 1:**
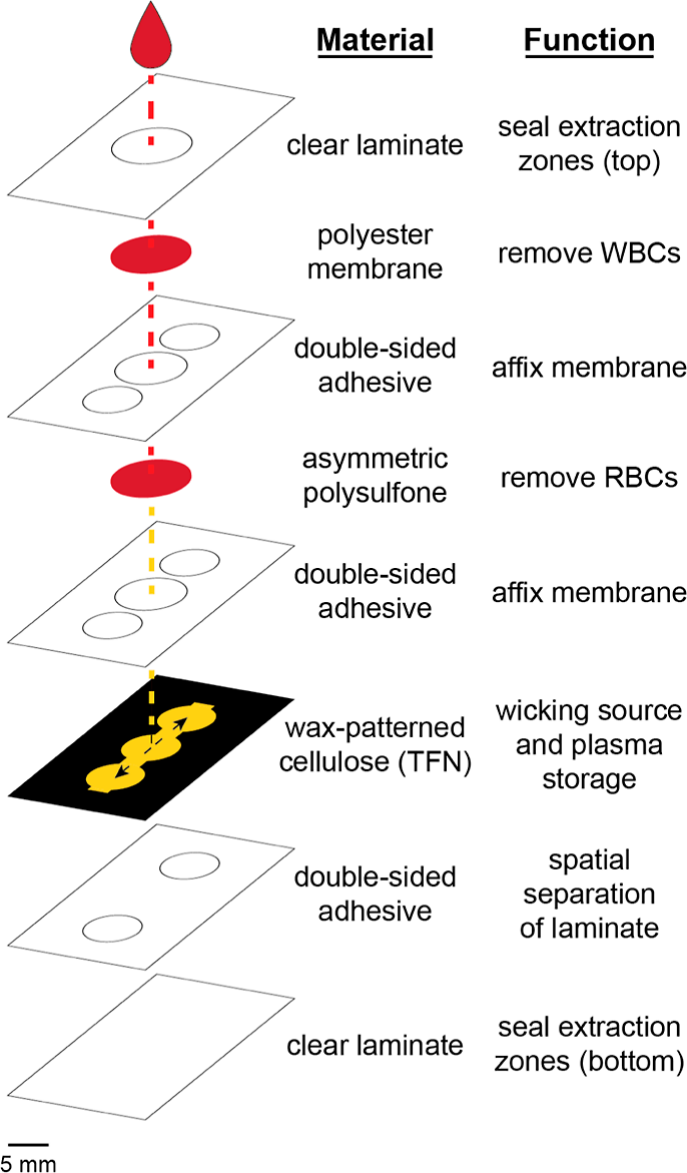
Exploded schematic of the pDPS card. Whole blood is applied directly to the top, center zone of the card, which is defined by a port in a film of the plastic laminate. The blood wicks vertically through the polyester membrane and asymmetric polysulfone layers to remove WBCs and RBCs, respectively. Patterned cellulose provides a source of capillarity to laterally wick and store cell-free liquid plasma. Clear laminate layers on the top and bottom protect against concentrative evaporation in the collection zones during the drying process. All layered materials are held together using double-sided adhesive.

pDPS cards comprise three layers of porous materials with distinct functions: (i) fibrous polyester matrix to remove WBCs (Pall Leukosorb), (ii) asymmetric polysulfone membrane to remove RBCs and platelets (PSM; Pall Vivid GR), and (iii) wax-patterned cellulose for sample distribution and storage (Figure 1). The remaining double-sided adhesive and clear laminate layers maintain conformal contact between layers and protect against uneven evaporation, respectively. Leukosorb and Vivid GR PSM have a considerable void volume, which reduced the available liquid volume of the sample by approximately 50%.^37^ Therefore, the pDPS card only yielded two collection zones from the same sample input of 75 μL of whole blood in comparison to our previously reported pDBS card that yielded four zones of whole blood. Increasing the sample input and scaling the area of each separation membrane could increase the number of paper discs or volume of the plasma sample obtained from the card. However, the data presented in this manuscript maintains the World Health Organization (WHO) recommended sample input of 75 μL and output of a standard 6 mm-diameter office hole punch.^38^

We patterned the top and bottom of the cellulose layer with unique geometries to optimize sample volume and allow reproducible filling that is independent of the hematocrit (Figure S1).^39−41^ Whole blood is transported from the sample addition zone vertically through both membranes via capillary action and ultimately into the cellulose layer below. Cell-free plasma wicks along the lateral channels and is stored in two distinct collection zones. The two arms of the lateral channels are of equal length positioned the same distance away from the sample inlet to equalize flow.^42^ Spatially positioning the extraction zones at a distance from the separation materials serves two functions: (i) it permits visible inspection of plasma separation for filling and quality and (ii) minimizes sample contamination by liberated hemoglobin because the collection zones are not located directly beneath the separation membranes (Figure S2).

### Card Operation and Handling

We applied a discrete volume of blood (75 μL) to the center of the card directly onto the Leukosorb membrane with the aid of a volumetric pipette. After the addition of whole blood, pDPS cards passively separate plasma within 5 min, independent of the hematocrit. Controlling evaporation of liquid plasma in the cellulose layer is a critical component for producing consistent sample volumes in each 6 mm-diameter punch (e.g., limiting evaporative concentration). To achieve this control, we included removable laminate layers on the top and bottom of the pDPS card to maintain a simple user interface and allow the drying process to occur under ambient conditions (i.e., on a lab benchtop or in a biosafety cabinet). Following separation and drying of plasma (ca. 5 h), we used a pair of tweezers to remove the top and bottom protective laminate sheets. Then, we removed both collection zones at the ends of the lateral channel using a standard hole punch (6 mm-diameter) directly into individual sampling tubes containing an aqueous buffer solution that is compatible with downstream analysis.

### Card Calibration Scheme

Analytes may adsorb irreversibly to the various porous materials (i.e., Leukosorb, PSM, and TFN) that comprise the device, which could impact the accuracy of measurements made in comparison to liquid plasma (i.e., the reference method). Therefore, we calibrated the pDPS card using liquid standards to account for analyte adsorption and associated matrix effects when appropriate. We applied calibrants of known concentrations to the cards and allowed them to fully dry prior to elution and subsequent analysis. For comparison, we also analyzed matched concentrations of liquid calibrants in tandem to evaluate the extent of adsorption or analyte loss to the materials.

## Results and Discussion

### pDPS Cards Reproducibly Fill Independent of Hematocrit Values

**Figure 2:**
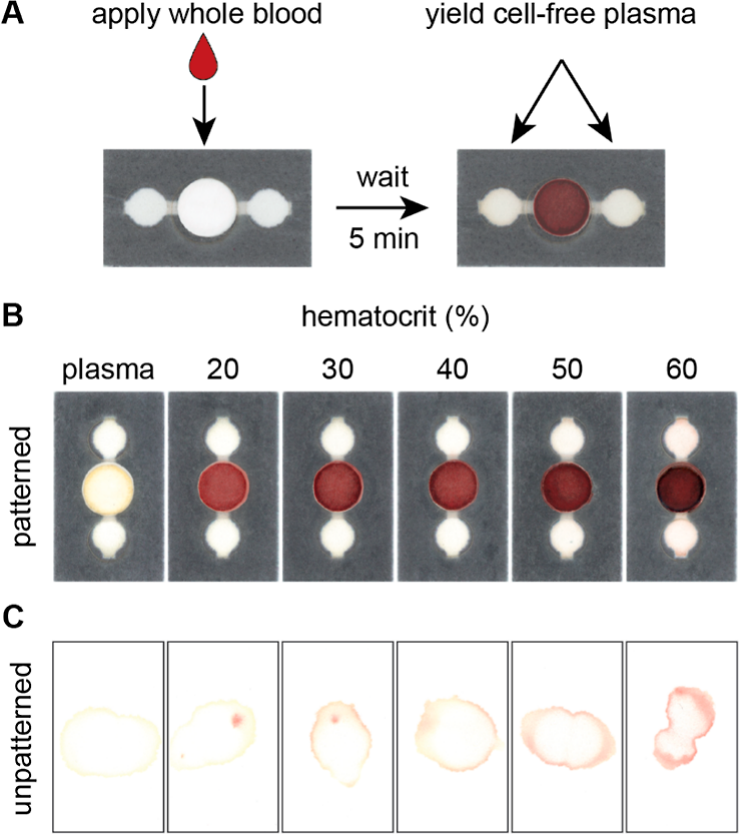
pDPS cards fill independent of the hematocrit with minimal hemolysis. (A) Image of a pDPS card before (left) and after (right) the addition of blood to the sample input zone. The sample addition zone located in the center of the card also permits cellular analysis and extraction of genomic nucleic acids. Representative images of (B) pDPS cards and (C) unpatterned TFN paper (membranes were removed following separation to reveal irregular dried plasma spots) across the physiological range of hematocrits (20–60%) highlight the benefit of directing plasma away from the separation materials to reduce contamination of collected plasma.

The yield of plasma obtained using porous materials for passive separation of blood is largely dependent on the intrinsic properties of the blood sample (e.g., hematocrit value) and ambient conditions during separation and drying (e.g., extent of evaporation, which is a function of ambient temperature and relative humidity). Blood samples with a lower hematocrit (e.g., 20%) are expected to have a theoretical plasma volume of 60 μL for a sample input volume of 75 μL. In contrast, blood samples with a higher hematocrit (e.g., 60%) will only have a theoretical plasma volume of 30 μL for the same input sample volume of 75 μL. Therefore, a plasma separation device must be capable of metering excess liquid plasma from samples with a lower hematocrit, while also completely saturating the collection zones when the hematocrit is high. To achieve this broad usability, we patterned the paper layer of pDPS cards with hydrophobic wax barriers to constrict fluid flow and guide sample distribution to the collection zones at the end of the lateral channel and away from the sample addition zone (Figure 2A). In this format, pDPS cards fill reproducibly using a single sample input volume of 75 μL across the physiological range of hematocrit values (20–60%, Figure 2B). However, applying the same samples of blood to unrestricted and undefined collection papers (i.e., unpatterned TFN) yielded irregular shaped dried plasma spots with increased susceptibility for contamination due to hemolysis visualized by red spotting on the TFN (Figure 2C). Restricting the amount of porous material available for wicking plasma provided reproducible, discrete volumes of plasma without user intervention.

The second factor—extent of evaporation—was responsible for consistently yielding a discrete volume of liquid plasma independent of changes under ambient conditions (i.e., temperature and humidity). If the punch zones remain exposed to the environment (i.e., unsealed), then evaporation can effectively concentrate soluble analytes by driving excess liquid plasma to the punch zones.^43^ We demonstrated this effect by quantifying the soluble transferrin receptor across the physiological range of hematocrits. Utilizing blood from a single donor and adjusting the hematocrit by the addition or subtraction of native plasma allowed direct comparison of measurements between two configurations of pDPS cards. In the unsealed device, all samples yielded a higher concentration of the transferrin receptor (average 77.2 ± 15.5 ng/mL) compared to the liquid reference samples (average 54.0 ± 2.9 ng/mL) (Figure S3A). In contrast, the sealed device yielded overall lower concentrations of the transferrin receptor (average 20.4 ± 2.4 ng/mL) compared to the liquid reference samples (average 51.9 ± 2.8 ng/mL) (Figure S3B). We identified one data point as an outlier from the sealed device data using Grubbs’ test (alpha = 0.05), which was removed. Although the unsealed device produced a more accurate average concentration with respect to the percent error across hematocrits compared to the sealed device (+43% vs −61% error, respectively), the coefficient of variation (CV) was improved using the sealed device (20.1% vs 11.8% CV, respectively). The unsealed device had a difference of 32.5 ng/mL between the lowest and highest concentrations (measured from 30 and 60% hematocrit, respectively). In stark contrast, the sealed device only varied by 6.3 ng/mL across all hematocrit values, representing a fivefold improvement compared to the unsealed device. We performed a one-way ANOVA to compare the effect of five different hematocrit values on the quantitation of transferrin receptor protein from pDPS cards, which revealed that there was not a statistically significant difference between mean transferrin receptor protein concentrations for whole blood samples with varying hematocrit values (*F* = [1.893] and *p* = 0.15). The overall yield of analytes eluted from TFN can be improved by introducing low concentrations of surfactants into the elution buffer to encourage the resolubilization of proteins following the drying step (vide infra).^44^ However, maintaining a consistent concentration of this soluble analyte provided confidence in the volume metering capabilities of this device. Additionally, reproducibly producing a discrete volume of liquid plasma—independent of the hematocrit—can enable quantitative analysis to aid in a clinical diagnosis rather than simply detecting the presence of an analyte.

### Generation of Discrete Plasma Volumes with No Evidence of Hemolysis

After we altered the device with laminate sheets to control evaporation, we calibrated the device with hemoglobin standards to estimate the volume of liquid plasma contained in a single 6 mm-diameter hole punch according to a previously described method.^35^ Because it comprises ≥95% of the dry weight of an RBC,^45^ liquid hemoglobin standards provide a measurable quantity of analytes that can wick through the assembly of porous separation materials to simulate the fluid flow of a sample of whole blood. Briefly, we calibrated pDPS cards using liquid hemoglobin standards across the physiological range of concentrations (3–18 g/dL) and fit the data using linear regression (Figure S4). Then, we input the resultant slope into a previously established relationship between the calibration slope and known input sample volume (eq 1), where *y* is the slope of the linear regression for calibrated pDPS cards and *x* is the volume of the sample in microliters.^35^
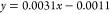
1

Using this relationship, we estimated that each 6 mm-diameter paper disc contained a volume of 8.6 ± 0.7 μL of liquid hemoglobin (total volume approximately 17.2 μL per card). The calculated theoretical volume occupied in a 6 mm-diameter paper disc of grade TFN (ca. 470 μm thick with a nominal porosity of 80%) is approximately 10.6 μL. Although the pDPS card was fabricated from the same TFN cardstock as our previously reported pDBS card, it contains approximately 1.8 μL less sample in the same 6 mm-diameter paper disc using identical methods. We attributed this difference to the protective laminate sheets used to seal the hydrophilic features of the card, where the surfaces responsible for wicking plasma are exposed to a hydrophobic material rather than air.

Impurities in recovered plasma may arise from the release of intraerythrocytic analytes such as hemoglobin and potassium. RBCs can be lysed by shear forces caused from overloading the PSM (e.g., excess number of RBCs due to a higher sample volume or high hematocrit). We demonstrated the purity of plasma samples produced by the pDPS card by quantification of hemoglobin across a wide range of hematocrits (20–60%). Visually, a slight pink hue can be observed at the periphery of the scanned image of the pDPS card with a blood sample at 60% hematocrit (Figure 2B,C). Although the presence of a pink coloration in the collection zone can be indicative of hemolysis, all plasma samples eluted from the pDPS cards produced hemoglobin concentrations that fell below the limit of detection (LOD, 0.17 g/dL) of our assay. Similarly, the reference plasma sample obtained via centrifugation also fell below the LOD. Therefore, the extent of hemolysis can be considered negligible and should not significantly impact downstream quantitative methods. We further evaluated the utility of plasma obtained from pDPS cards for quantitation of a low abundance protein (transferrin receptor), a specific ion (potassium), and nucleic acids from the center punch (18S rRNA).

### Quantitative Elution of a Low Abundance Protein

**Figure 3:**
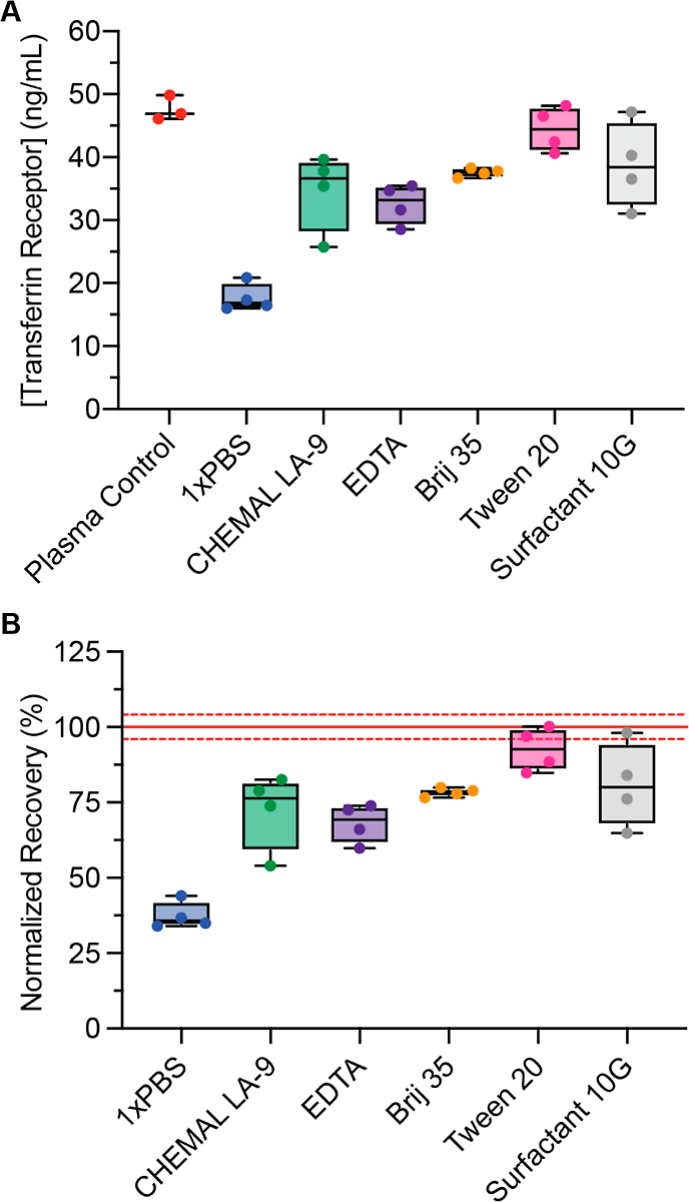
Quantitation of soluble transferrin receptor protein by ELISA. (A) Various surfactants (0.05% v/v) were investigated for quantitative elution of the transferrin receptor from TFN paper (*N* = 4 per condition and *N* = 3 for plasma control). (B) Recovery of the transferrin receptor was normalized as a percentage of the average concentration of the transferrin receptor in a reference liquid plasma sample (48.7 ± 1.4 ng/mL, *N* = 3), which is indicated by a solid red line with the error at a 95% confidence interval (dashed red lines). Each elution condition was performed in quadruplicate.

Accurate diagnosis and proper treatment of a disease typically requires quantitation of a specific biomarker rather than its simple detection. Clinical reference values serve to guide diagnosis, but loss or degradation of the analyte of interest could result in errors. For example, myriad proteins are present in plasma at low concentrations,^46^ which could be obscured or highly attenuated by nonspecific adsorption to the porous materials in the device. We quantified a low abundance protein—soluble transferrin receptor (ca. ng/mL)—using an ELISA to evaluate the extent of nonspecific adsorption of protein to pDPS cards (Figure 3A). Our initial investigations yielded approximately 44% recovery of the transferrin receptor protein using only 1X phosphate-buffered saline (PBS) as the eluent (Figures 3B and S3B). Typically, elution buffers are formulated with a surfactant to encourage solubilization of analytes and reversal of adsorption to solid-phase surfaces; therefore, we screened five surfactants to achieve quantitative elution. We observed that each surfactant increased the recovery of transferrin receptor, where 0.05% Tween 20 yielded a nearly quantitative recovery (±10% of reference sample). Two-tailed Student’s *t*-test (C.I. 95%) yielded a *p*-value of 0.17, providing no evidence of a difference in the concentration of transferrin receptor protein between the two sets of plasma samples (liquid and dry). This supports the fact that the quality of our plasma sample was conserved even for a low abundance protein.

### Quantitation of a Specific Ion

**Figure 4:**
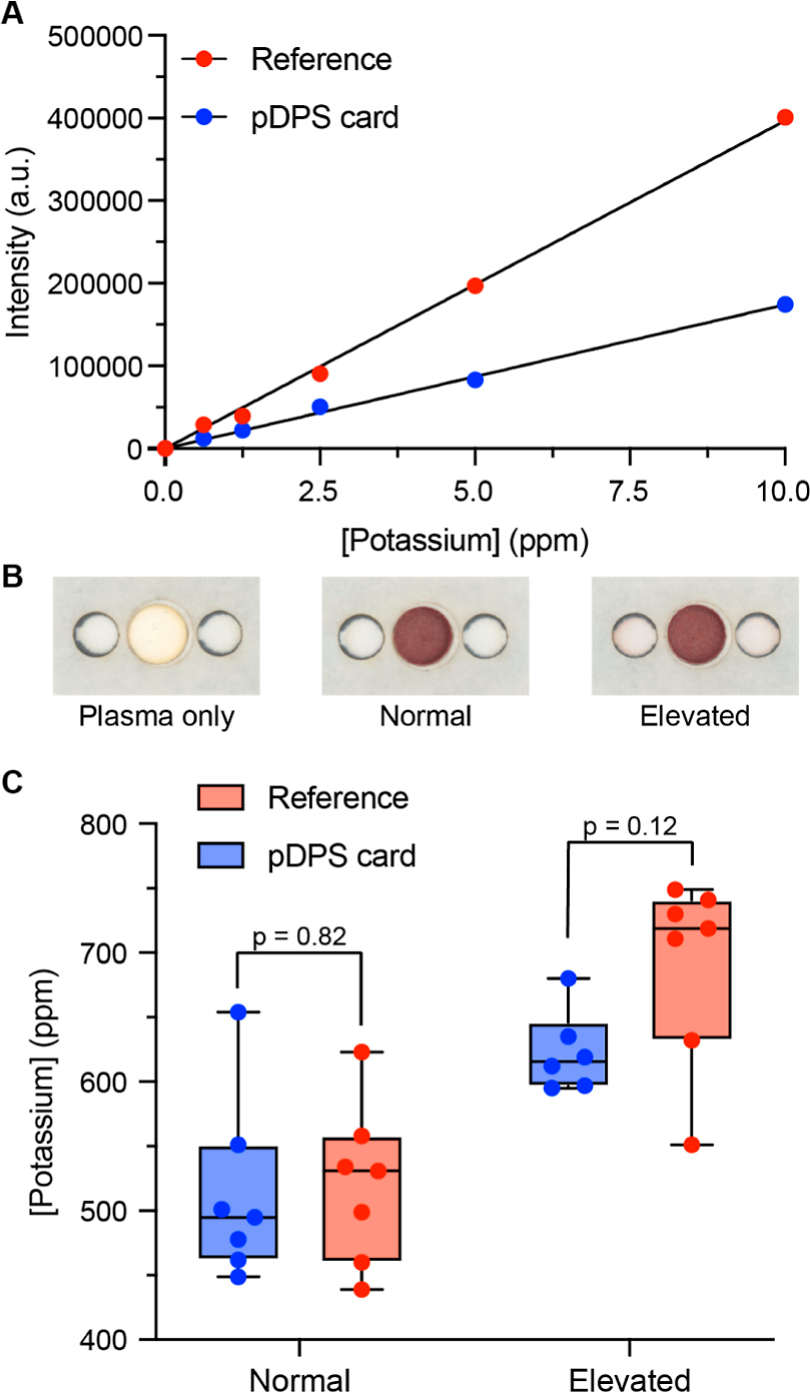
Quantitation of potassium by ICP-AES. (A) Individual calibration curves for the reference method (red marker; liquid sampling) and pDPS cards (blue marker; paper sampling). Each curve comprises matched liquid potassium standards (reference: *R*^2^ = 0.999, slope = 39701, and *y*-intercept = 0; *N* = 1; pDPS card: *R*^2^ = 0.998, slope = 17423, and *y*-intercept = 0; *N* = 2). (B) Representative images of pDPS cards with cell-free plasma, whole blood (normal potassium level), and spiked whole blood (elevated potassium level). Visual inspection of the collection zones supports the lack of hemolysis following separation and drying. (C) Concentration of normal and elevated levels of potassium in whole blood sampled using liquid reference and pDPS card methods (*N* = 7). A two-tailed Student’s *t*-test yielded *p*-values (C.I. 95%) of 0.82 and 0.12 for normal and elevated levels of potassium, respectively.

Extracellular potassium (i.e., serum or plasma) comprises only 2% of total body potassium, with the remaining 98% residing intracellularly.^47^ Even slight hemolysis of RBCs during separation can introduce massive quantities of potassium, resulting in erroneously elevated concentrations of potassium in plasma. Therefore, quantifying potassium by ICP-AES also served as a secondary method for evaluating the extent of hemolysis in addition to quantification of hemoglobin. Direct elution of two paper discs with water yielded only 35% recovery of potassium compared to matched liquid reference plasma obtained via centrifugation (Figure S5A). To elucidate whether the considerable loss of potassium was due to incomplete elution from the paper matrix, we performed complete digestion of the dried paper discs with concentrated nitric acid. This extraction method yielded approximately 44% recovery of potassium (Figure S5B). Due to the modest increase in recovery upon complete digestion, we attributed loss to adsorption of potassium to the separation membranes (e.g., PSM and Leukosorb). Since the membranes are responsible for filtering the cellular components of blood, further elution or digestion of the membranes would result in release of intraerythrocytic potassium and could artificially increase the concentration of potassium in the sample. Instead, we calibrated the pDPS cards with liquid potassium standards to account for (i) the associated loss to the materials and (ii) nonquantitative elution from the paper discs (Figure 4A). Comparison of the linear regression values obtained from both liquid and pDPS samples supported the measured loss of potassium of approximately 56%, representing a recovery of 44% as observed for fully digested paper discs. Both liquid and paper calibrations demonstrated excellent linearity over the physiological range of potassium.

We applied samples of whole blood (normal) and potassium-spiked whole blood (elevated) to pDPS cards to evaluate the quantitation of potassium in comparison to liquid reference samples. For visual comparison, we added liquid plasma from these samples, which we prepared via centrifugation, to pDPS cards. Following separation and drying of blood, we observed no visible signs of hemolysis for normal or elevated potassium levels in whole blood (Figure 4B). We identified one data point as an outlier in the elevated potassium range in pDPS card data using Grubbs’ test (alpha = 0.05), which was removed. The concentration of potassium calculated for both the normal and elevated pDPS samples was 513 ± 71 ppm (14% CV) and 623 ± 31 ppm (5% CV), respectively (Figure 4C, *N* = 7). Similarly, the concentration of potassium calculated for both the normal and elevated liquid reference plasma samples was 521 ± 62 ppm (12% CV) and 690 ± 73 ppm (11% CV), respectively. These data represent 1.6 and 9.7% error for the quantitation of normal and elevated potassium, respectively, using pDPS cards. A two-tailed Student’s *t*-test (C.I. 95%) yielded *p*-values of 0.82 and 0.12 for normal and elevated potassium, respectively, both indicating no evidence of a difference in the quantitation of potassium using pDPS cards compared to using liquid plasma prepared via centrifugation.

Finally, we compared the concentration of potassium in each sample to the expected clinical values. If plasma samples were experiencing even low levels of hemolysis during separation and drying, then the potassium levels in samples obtained with pDPS cards would be significantly higher than the liquid plasma samples obtained via centrifugation. Clinically, the concentration of potassium is reported in millimolar (mM), where the expected normal range is 3.5–5.0 mM and elevated range is >5.5 mM.^48^ The normal and elevated potassium concentration in our liquid plasma samples fell within the expected ranges at 5.2 ± 0.6 mM and 6.8 ± 0.7 mM, respectively. Accurately differentiating between normal and elevated potassium concentrations is critical for appropriate diagnoses and subsequent treatments. A two-tailed Student’s *t*-test (C.I. 95%) yielded a *p*-value of 0.0048, which supports the fact that normal and elevated levels of potassium are differentiable using pDPS cards. Maintaining appropriate concentrations of potassium following separation provides additional support for the superior quality of plasma generated by our cards.

### Extraction of Nucleic Acids from the Separation Materials

Promoting the separation of the cellular and liquid components expands the usability of a single blood sample. Plasma remains the gold standard for immunodiagnostic tests, ion quantification, viral load assessment, and protein analysis for metabolomic studies,^49^ while the cellular fraction can be used for nucleic acid analysis, from the patient and/or an infectious agent.^50^ Nucleic acid analysis of pathogens does not suffer from the common drawbacks of antibody-based diagnostic testing (e.g., inability to discriminate an active infection from past infections).^49^ We chose to detect and quantify by qPCR the 18S ribosomal RNA to demonstrate the recovery of intact nucleic acids from the dried cellular fraction from the blood inlet zone of the pDPS card, owing to this biomarker’s versatility: it is present in all eukaryotic cells, a well-established marker for organismal classification,^51^ used diagnostically (e.g., *Candida*(49) and *Plasmodium*(52)), and a common internal normalization gene (housekeeping gene) for RT-qPCR.^53^

**Figure 5:**
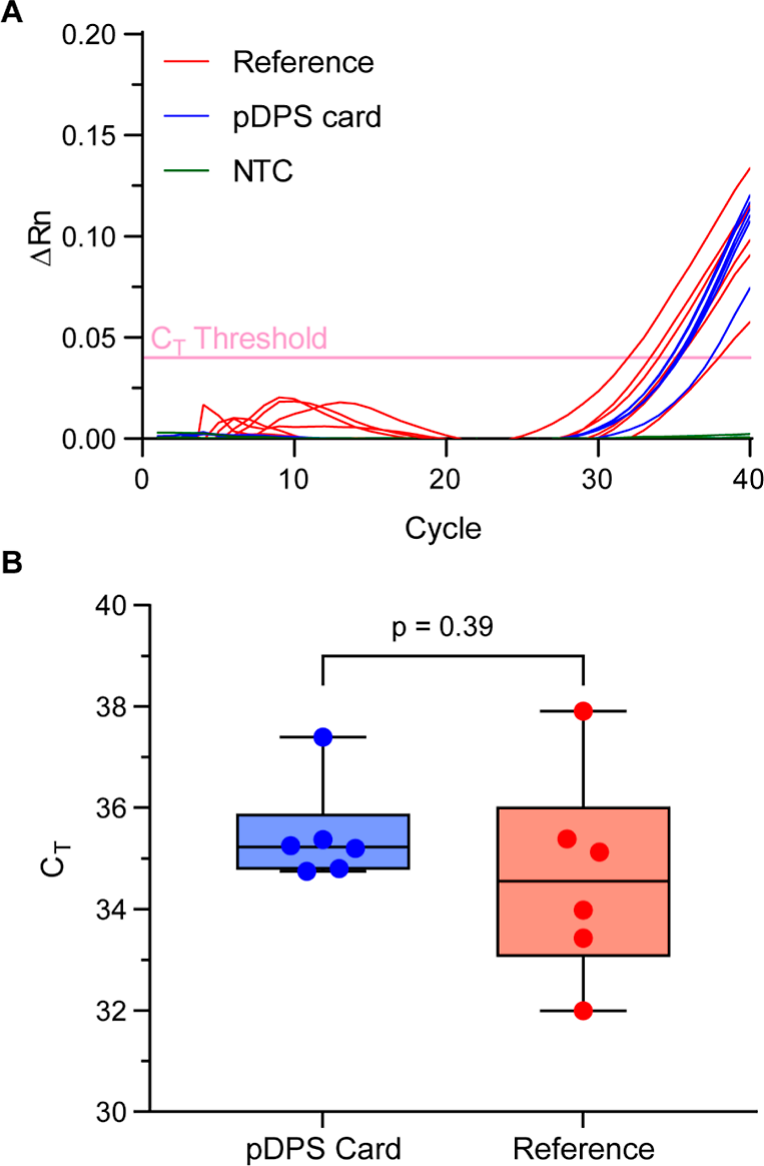
18S rRNA amplification from white blood cells contained in the pDPS sample inlet layers. (A) Amplification plots for the reference method (whole blood liquid sampling, red trace, *N* = 6), pDPS central punch (paper sampling, blue trace, *N* = 6), and no template control (NTC, green trace, *N* = 3). (B) Threshold cycle (*C*_T_) comparison between pDPS central punch (paper sampling, blue plot, *N* = 6) and reference method (whole blood liquid sampling, red plot, *N* = 6) for the amplification plots presented in (A). A two-tailed Student’s *t*-test (C.I. 95%) yielded a *p*-value of 0.39, indicating that there is no statistically significant difference between nucleic acid extracts from the reference method and pDPS cards.

We applied 75 μL of whole blood to pDPS cards to evaluate the extraction of DNA from a dried specimen, via measurement of the amplification cycle threshold (*C*_T_) of 18S rRNA by RT-qPCR, in comparison to an equal volume of liquid whole blood (Figure 5A). The single peak in the melting curves for both liquid samples and punches from pDPS cards (Figure S6A), as well as single bands at 63 bp in the agarose gel (Figure S6B), indicate that the chosen primers were specific for a region of 18S rRNA. *C*_T_ values from reference liquid samples and pDPS cards were 35 ± 2 cycles (6% CV) and 35 ± 1 cycles (3% CV), respectively (Figure 5B). A two-tailed Student’s *t*-test yielded a *p*-value of 0.39, indicating no statistically significant difference between the extraction and amplification of nucleic acids using the pDPS blood inlet zone and liquid whole blood. These results demonstrate a substantial advantage that stems from the design of the pDPS card: the consistency of the sampled area, both for paper discs and the blood inlet zone, reproducibly meters volumes and enables quantitation of analytes. The 10 mm membrane discs (Leukosorb and Vivid GR PSM) contain all cellular matter present in the sample, and both membranes are used integrally to eliminate sampling bias.

## Conclusions

We aimed to expand the testing capabilities of biofluids at the point-of-care by designing a patterned card for separating and storing plasma from fingerstick volumes of whole blood. Our approach combined the enhanced sampling afforded by controlling the flow and distribution of biofluids within the porous matrix and efficient separation of plasma from the cellular component of blood, which are both achieved without user intervention. pDPS cards operate autonomously following the addition of approximately 75 μL of blood to the top of the device. Spatially separating the plasma collection zones at the ends of lateral channels away from the separation materials produced high-quality, cell-free plasma with no evidence of contamination from intraerythrocytic analytes (e.g., hemoglobin or potassium). In this configuration, the plasma zones are easily viewed from the top of the device for visual inspection of the separation process. Appearance of the red or pink color in the sample collection zones would indicate that a severely hemolyzed sample was added and that the device should be discarded. Sealing the device with layers of clear laminate on the top and bottom protected against uneven evaporation and produced discrete volumes of plasma independent of the hematocrit value.

Quantification of three classes of analytes indicated the analytical quality of the purified plasma, and enriched whole blood samples are conserved in comparison to liquid reference samples (either plasma obtained via centrifugation or whole blood). Specifically, we achieved quantitative recovery of the soluble transferrin receptor protein with the addition of a low concentration of the surfactant in the elution buffer to liberate the proteins from the paper matrix following drying. Similarly, we calibrated pDPS cards with potassium standards to account for adsorption of potassium ions to the separation materials. Using this method of quantitation, we demonstrated superior quantitation of potassium with no evidence of a statistically significant difference compared to liquid plasma samples at normal and elevated concentrations. Finally, we extended the sampling capacity of pDPS cards by utilizing the sample addition zone (containing RBCs and WBCs) for quantifying 18S rRNA by RT-qPCR with no statistically significant difference between the extraction and amplification of nucleic acids from pDPS blood inlet materials and an equal volume of liquid whole blood. In addition to amplification of endogenous nucleic acids, we anticipate that the cellular fraction that is isolated and enriched in the center punch of a pDPS card can be used, for example, for genotyping HIV^50^ or immunophenotyping WBCs.^54^

This specific device geometry affords the microsampling of low volumes of blood to generate multiple sample compositions including blood cells (separation materials) and cell-free plasma (porous collection material). Spatially separating the collection zones at the ends of the lateral channel could allow different treatments to be applied to the card to enable stabilization of different analytes (e.g., proteins vs nucleic acids). We anticipate that maintaining a simple user interface for sample application and spatially separating the collection zones away from the separation materials will provide higher quality plasma samples at the point-of-collection, improve the accuracy for diagnostic applications in the laboratory, and lead to improvements in patient care.

## Supporting Information Available

Materials and methods, detailed schematic of the patterned TFN layer, representative images of pDPS cards after removing separation materials, controlled evaporation improves quantification of soluble transferrin receptor protein, estimation of the sample volume in pDPS cards, effect of the recovery method from pDPS cards for quantitation of potassium, analysis of amplicons from 18S rRNA from white blood cells, and primers used in RT-qPCR (PDF)
