# Supplementary material for: Direct Processing and Storage of Cell-Free Plasma Using Dried Plasma Spot Cards
Source: ACS Meas Sci Au. 2022 Jul 25;2(5):457–65. doi: 10.1021/acsmeasuresciau.2c00034 (PMC9585636; doi:10.1021/acsmeasuresciau.2c00034)
Supplement: Supplementary file 1 — tg2c00034_si_001.pdf [file tg2c00034_si_001.pdf]

Supporting Information

Direct Processing and Storage of Cell-free Plasma using Dried Plasma Spot Cards

Keith R. Baillargeon, Giorgio Gianini Morbioli, Jessica C. Brooks, Philip R. Miljanic, and Charles R. Mace*

Department of Chemistry, Laboratory for Living Devices, Tufts University, Medford, MA 02155, United States

* corresponding author email: charles.mace@tufts.edu

Pages: 16

Figures: 6

Tables: 1

Contents: Materials and Methods. Detailed schematic of patterned TFN layer. Representative images of pDPS cards after removing separation materials. Controlled evaporation improves quantification of soluble transferrin receptor protein. Estimation of sample volume in pDPS cards. Effect of recovery method from pDPS cards for quantitation of potassium. Analysis of amplicons from 18S rRNA from white blood cells. Primers used in RT-qPCR.

Materials and Methods

Chemical Reagents and Materials

We purchased Drabkin's reagent, Brij 35 (30% w/w), potassium ICP standard (1000 ppm in 3% nitric acid), and ACS reagent grade (ASTM Type I) water from Ricca Chemical. We purchased concentrated nitric acid (70% v/v), phosphate buffered saline, ethylenediaminetetraacetic acid, ethanol (200 proof), and QED Bioscience Surf's Up® surfactant kit (containing CHEMAL LA-9, Brij 35, Tween 20, surfactant 10G) from Fisher Scientific. We purchased lyophilized hemoglobin standard from Pointe Scientific (Canton, MI). We obtained a transferrin receptor protein quantification enzyme immunoassay kit (CAT: TFC-94) from Ramco Laboratories (Stafford, TX). We obtained samples of whole blood collected in sodium citrate vacutainers from Research Blood Components (Watertown, MA). We purchased Munktell TFN grade cellulose paper from Laboratory Sales & Service LLC (Somerville, NJ). We purchased Critoseal vinyl plastic putty from VWR. We purchased 40-mm microhematocrit capillary tubes from LW Scientific. We purchased Fellowes and Avery laminates from Amazon. We purchased ¼" clear acrylic sheets from McMaster-Carr. We purchased Vivid PSM grade GR and Leukosorb from Pall Corporation. We purchased a SuperScript™ III Platinum SYBR Green One-Step qT-qPCR Kit from ThermoFisher Scientific. We purchased a QIAGEN DNA Mini Kit from QIAamp. We purchased h18S rDNA primers from Integrated DNA Technologies (IDT). We purchased 1.2% Agarose gels from Lonza Bioscience.

Live Subject Statement

We obtained samples of human whole blood from healthy donors supplied by Research Blood Components (Watertown, MA). The vendor follows American Association of Blood Banks guidelines for all donors, which includes IRB approved consent to the use of collected blood for research purposes. All research was approved by the Tufts University Institutional Biosafety Committee.

Measurement and Adjustment of the Hematocrit

We measured the initial hematocrit of the whole blood sample upon arrival from the vendor. We added 3 µL of whole blood to a 40-mm microhematocrit capillary tube and sealed the tube at one end with Critoseal putty. We centrifuged the microhematocrit capillary tubes at 1,200 RPM for 3 minutes using a ZipCombo centrifuge from LW Scientific. We obtained images of the microhematocrit tubes using an 8-bit EPSON Perfection V600 PHOTO scanner with a resolution of 800 DPI. We calculated the hematocrit of the sample by measuring the ratio of the length that RBCs occupied in the tube to the total sample length with ImageJ software.[1] We followed the same procedure for each sample for the measurement of hematocrit (N=2)

We created samples of whole blood at different hematocrit values (20–60%) by adjusting the volume of native plasma in the sample. We confirmed the hematocrit value by measuring the hematocrit value as described above (N=2).

Fabrication of pDPS Cards

We used Adobe Illustrator to design the features of the card and printed the hydrophobic wax barriers using a previously reported double-sided transfer method.[2] Using this double-sided transfer method allows for unique features to be printed on the top and bottom of a single sheet of paper. First, we printed the top and bottom designs onto laminate sheets using a Xerox ColorQube 8580 wax printer. Next, we aligned a sheet of chromatography paper with the top and bottom designs using a custom acrylic alignment jig. Finally, we used a Promo Heat CS-15 T-shirt press (45 seconds at 280 °F) to transfer the wax from the laminate sheets to the paper to form hydrophobic barriers through the full thickness of the paper. These barriers control sample flow and distribution in pDPS cards. We cut all adhesive and laminate layers using a Boss laser cutter. We manually cut the PSM and Leukosorb membranes cut using a hammer-driven circular metal punch (10-mm diameter).

Estimation of Output Plasma Volume by Quantitation of Hemoglobin

We estimated the volume of plasma contained in each 6-mm diameter paper punch generated with pDPS cards by quantitation of hemoglobin using Drabkin's reagent and a standard protocol.[3] We constructed individual calibration curves using lyophilized hemoglobin standards rehydrated in ACS reagent grade (ASTM Type 1) water over a range of 3–18 g/dL. We varied the input sample volume of hemoglobin standard used for each calibration curve (3–11 µL) and kept the diluent volume constant (1 mL Drabkin's reagent). We applied 75 µL of the same liquid hemoglobin standards to pDPS cards (N=3 cards per concentration) to simulate the flow of blood in the device. Samples dried overnight in a biosafety cabinet (*ca.* 16 hours) before analysis. We used a standard office hole punch to remove a 6-mm diameter punch from each end of the lateral channel containing cell-free plasma (N=2 replicate punches per card). Then, we submerged both punches in 1.0 mL Drabkin's reagent for 30 minutes before quantifying the concentration of hemoglobin (pDPS and liquid samples) in a 96-microwell plate with a Varioskan LUX microplate reader at 540 nm wavelength. We performed linear regression for each calibration curve using GraphPad Prism. We defined the limit of detection (LOD) as the lowest average analyte concentration that could be distinguished from the blank plus three standard deviations.[4] Our blank sample was the Drabkin's assay solution without hemoglobin present (N=20) and our low concentration sample was pure plasma obtained via centrifugation from three different donors (N=20).

Quantification of Soluble Transferrin Receptor Protein by ELISA

We quantified the concentration of soluble transferrin receptor protein in human plasma using a commercially available ELISA kit from Ramco Laboratories. Briefly, we added 50 µL of each sample, control, and standard into replicate wells in a 96-well plate. Then, we added 150 µL horseradish peroxidase-conjugate to each well, sealed the top of the plate, mixed at room temperature for 10 minutes (190 RPM on shaker), and incubated at room temperature for 2 hours.

Following incubation, we washed the plate thrice and added 200 µL substrate solution. Next, we mixed at room temperature for 1 minute (190 RPM on shaker) and incubated in the dark for 30 minutes. Finally, we added 50 µL stop solution into each well, mixed for 1 minute (190 RPM on shaker), and measured absorbance using a Varioskan LUX microplate reader at 450 nm wavelength. We prepared the following samples for analysis: (i) dried plasma from pDPS cards (75 µL input whole blood per card), (ii) liquid plasma via centrifugation (5 min at 800×g), (iii) transferrin receptor protein controls (provided in kit), and (iv) calibration standards (provided in kit). For dried plasma samples from pDPS cards, we removed two punches (6-mm diameter) and submerged them directly in the supplied diluent (1.4 mL). Similarly, we diluted an equivalent volume of liquid plasma reference samples (17.2 µL) and controls in the supplied diluent (1.4 mL). Blank samples comprised deionized water. For elution studies, we followed the above protocol with the following change: we varied the concentration of surfactant (final concentration 0.05% v/v) in phosphate buffered saline instead of using the supplied diluent. We investigated four surfactants (Tween 20, Brij 35, CHEMAL LA-9, and surfactant 10G) and EDTA for achieving optimal elution of transferrin receptor protein.

Preparation of pDPS Samples for the Quantitation of Potassium by ICP-AES

We applied 75 µL of venous whole blood to pDPS cards and allowed them to dry overnight. We pooled two punches (6-mm diameter) from each pDPS card and prepared liquid plasma reference samples via centrifugation. We added both samples (pDPS punches and liquid reference) to individual 5 mL round bottom flasks and digested the samples in 3 mL nitric acid (70% v/v) at 100 °C for 90 minutes. Following digestion, we evaporated the remaining nitric acid from the round bottom flasks. Once cooled, we reconstituted the samples in 3 mL nitric acid (10% v/v) and transferred the contents to 5 mL Eppendorf tubes. We prepared blank samples with two punches (6-mm diameter) of unpatterned TFN and followed the digestion procedure above. We also eluted pooled paper punch samples from pDPS cards using 3 mL ACS reagent grade (ASTM

Type 1) water in 5 mL Eppendorf tubes at 4 °C overnight (*ca.* 16 hours). We calibrated the ICP-AES with liquid calibrants (0.3–10 ppm potassium) before each experimental run. We analyzed all digested and eluted samples at a wavelength of 766.5 nm using a single phase, high dispersion Prodigy Spec ICP-AES by Leeman Labs Inc. (Hudson, NH).

Preparation of samples for DNA extraction

We applied 75 µL of venous whole blood to 6 pDPS cards and allowed them to dry overnight. After drying, we peeled off the adhesive layers that kept Leukosorb and PSM membranes adhered to TFN, and we punched them out of the adhesive layers using a hammer-driven circular metal punch (11-mm diameter). We transferred the membranes (Leukosorb and PSM) to a 2-mL centrifuge tube, making sure the membranes touched the bottom of the tubes. We performed the extraction accordingly to the manufacturer's instructions, with no deviations from the protocol.[5] For whole blood samples (liquid reference), we added 75 µL of venous whole blood to 6 2-mL centrifuge tubes and performed the extraction following the manufacturer's instructions (QIAamp® DNA Mini and Blood Mini Handbook), with no deviations from the protocol. We added 125 µL of PBS 1X buffer to bring the sample up to the required 200 µL.

qPCR amplification of DNA extracts and gel electrophoresis

We used an Applied Biosystems QuantStudio 3 Real-Time PCR System to perform quantitative PCR (qPCR). We set reaction volumes as 50 µL/well, using the SuperScript™ III Platinum SYBR Green One-Step RT-qPCR Kit. Each reaction contained 16 µL of Nuclease-Free (NF) water, 25 µL of 2x SYBR green reaction mix, 1 µL of Superscribe III Taq Mix, 2 µL of Reverse and Forward primer mixture (5 µM in each primer, **Table S1**), 1 µL of ROX reference dye, and 5 µL of DNA sample extract. We used NF water as the negative control (No Template Control, NTC).

We set the amplification profile as a single cycle of enzyme activation at 95 °C for 20 s, followed by 40 cycles of denaturation at 95 °C for 1 s and annealing at 60 °C for 20 s, with single fluorescence acquisition. We set the melt curve profile as a single cycle at 95 °C for 1 s, followed by an annealing step at 60 °C for 20 s and a dissociation step at 95 °C for 1 s, with single fluorescence acquisition.

After amplification, we analyzed PCR products using a Lonza FlashGel System. The analysis was performed as per manufacturer's instructions. Briefly, we hydrated the wells using 500 µL of NF water, adding the water in the first well and tilting the plate, so excess water would flow from one well to the next. Next, we added 4 µL of 5X loading dye to 10 µL of sample and mixed it thoroughly by pipetting. We carefully added 4 µL of the stained sample to each well of a Lonza 1.2% Agarose gel, and 4 µL of DNA ladder markers to both outer wells. We ran the separation at 250 V for 6 min, using a BioRad PowerPac HC power supply, using a Lonza camera and dock system. We recorded the gel image after 6 min.

Figure S1. Detailed schematic of patterned TFN layer. The top and bottom sides of the TFN layer are patterned with unique designs to reduce the void volume of specific features (*e.g.,* the lateral channel and circular zone directly beneath the separation materials). Collection zones are positioned 8.6 mm on center from the center circular zone. The dimensions below represent the pre-melted features. After melting the wax to form hydrophobic barriers through the full thickness of the paper, the final dimensions of the internal features (*e.g.,* collection zones, lateral channel) are reduced by approximately 1 mm.

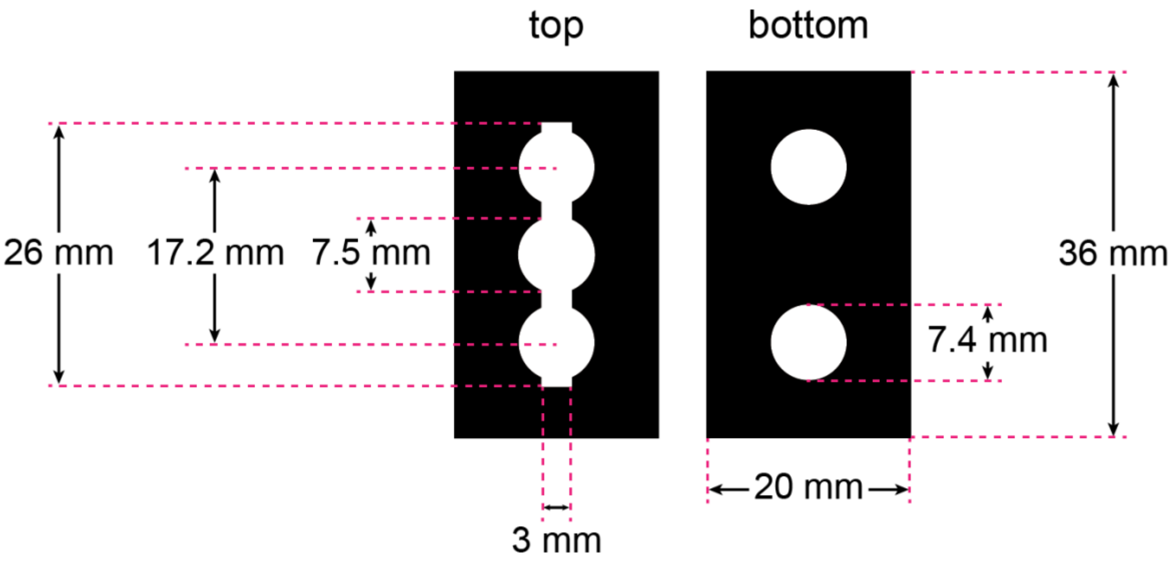

Figure S2. Representative images of pDPS cards after removing the separation materials to expose the paper layer below. Red spotting caused by hemoglobin liberated from lysed red blood cells can be observed directly beneath the separation materials (outlined in red) but not in the extraction zones located at the ends of the lateral channel.

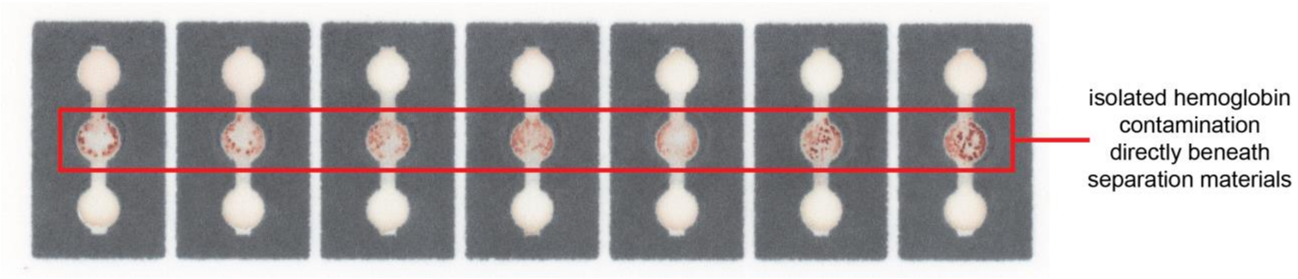

Figure S3. Controlling evaporation improves quantification of soluble transferrin receptor protein. Whole blood samples (20–60% hematocrit) were applied to (A) unsealed (no protective laminate) and (B) sealed (protective laminate on top and bottom of extraction zones) pDPS cards (N=5). Liquid plasma was produced as a reference sample via centrifugation.

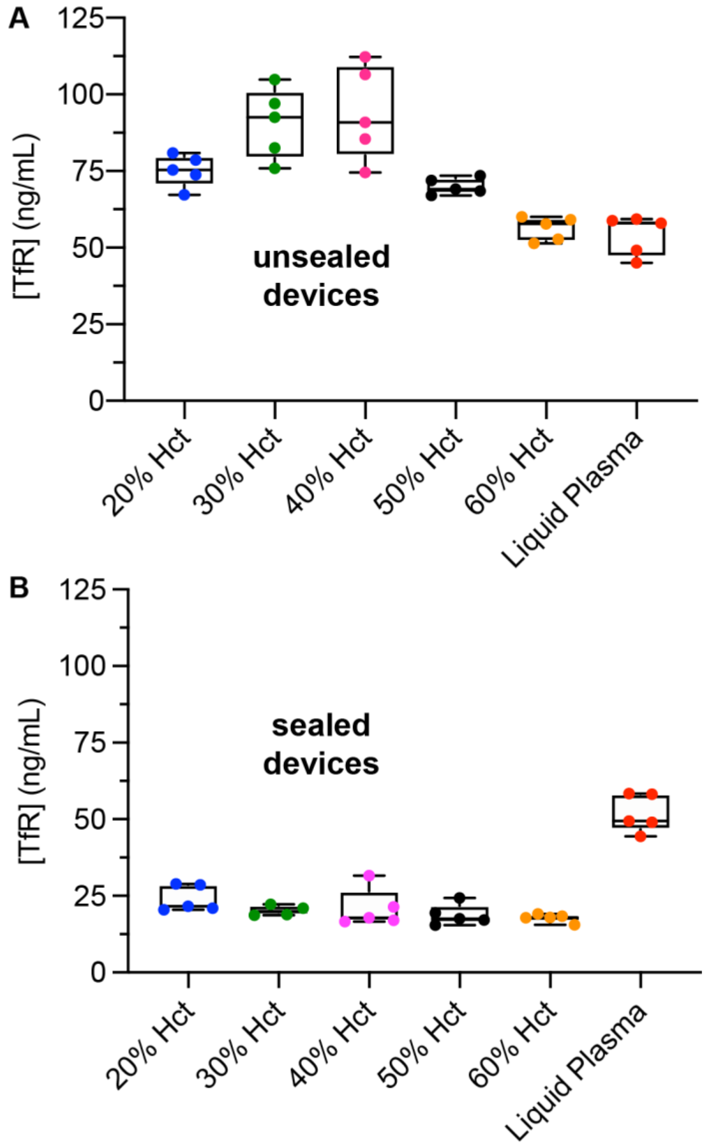

Figure S4. Estimation of sample volume in pDPS cards. pDBS cards were calibrated with liquid hemoglobin standards. The slope of the calibration curve (R^2 = 0.992, slope = 0.0257, y-intercept = -0.0544) was used to estimate the sample volume. Each data point represents the average of six replicates and error bars represent the standard error of the mean.

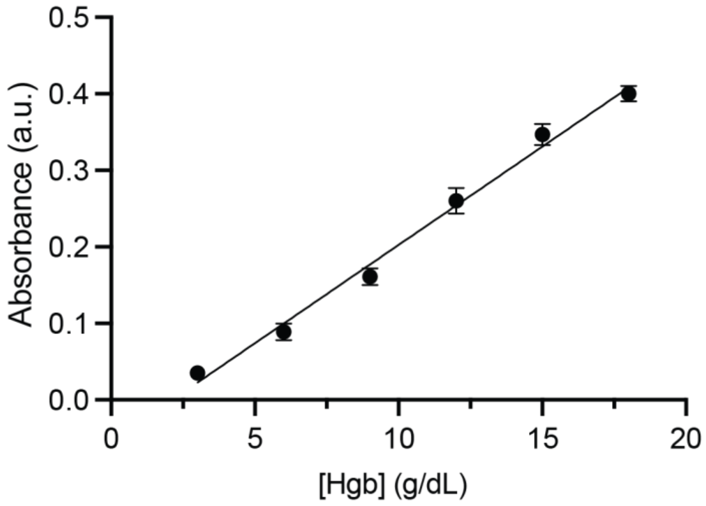

Figure S5. Effect of recovery method from pDPS cards for quantiation of potassium. (A) Initial quantitation of potassium eluted directly from dried pDPS cards using water as the eluent (N=20) yielded only 35% recovery compared to the liquid reference plasma (two-tailed Student's *t*-test (C.I. 95%; p-value < 0.0001). (B) Similarly, quantiation of potassium after complete digestion of dried pDPS cards by nitric acid (N=3) yielded only 44% recovery compared to digested liquid reference plasma (two-tailed Student's *t*-test (C.I. 95%; p-value = 0.0002).

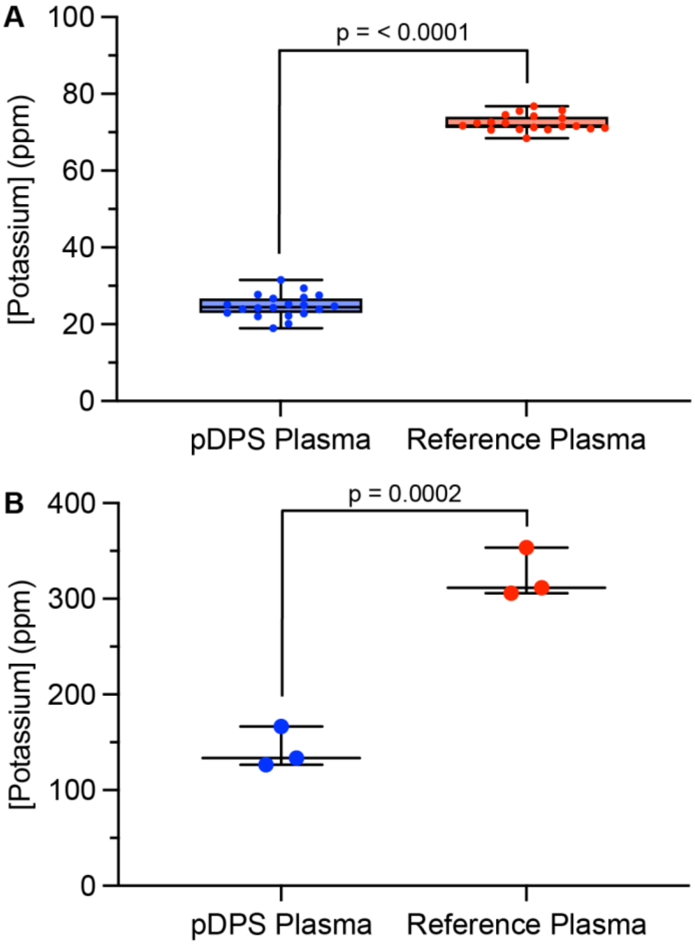

Figure S6. Analysis of the amplicons originated in the amplification of a 63-bp sequence from the ribosomal 18S rRNA from white blood cells. (A) Melting curves of the amplified nucleic acids extracts for reference method (whole blood liquid sampling, red trace, N=6), pDPS central punch (paper sampling, blue trace, N=6), and no template control (NTC, green trace, N=3). The single peak indicates that the primers were specific, with minimal primer dimer formation in PCR products. (B) Agarose gel analysis of PCR amplicon products presented in (A). Lanes: (1) DNA ladder; (2) and (3) No Template Control (NTC). (4)–(7) Amplicons from reference method extracts (63 bp). (8) Empty lane. (9)–(12) Amplicons from pDPS punches extracts (63 bp). (13) DNA ladder.

A

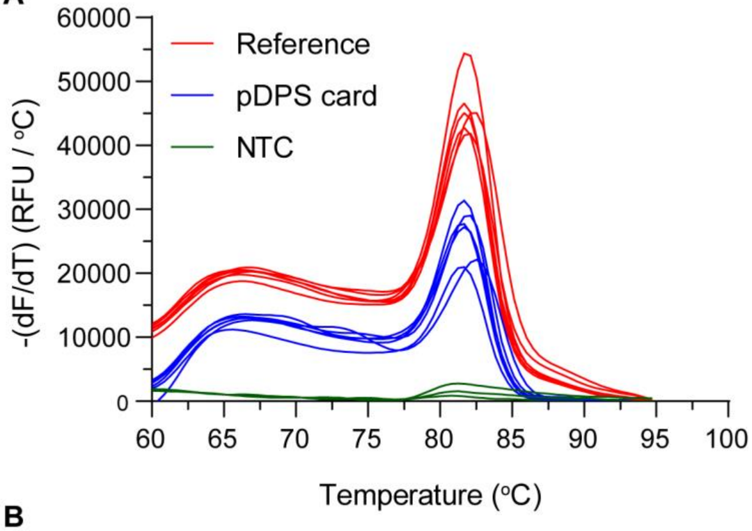

Reference
pDPS card
NTC
-(dF/dT) (RFU / °C)
Temperature (°C)
60000
50000
40000
30000
20000
10000
0
60
65
70
75
80
85
90
95
100

B

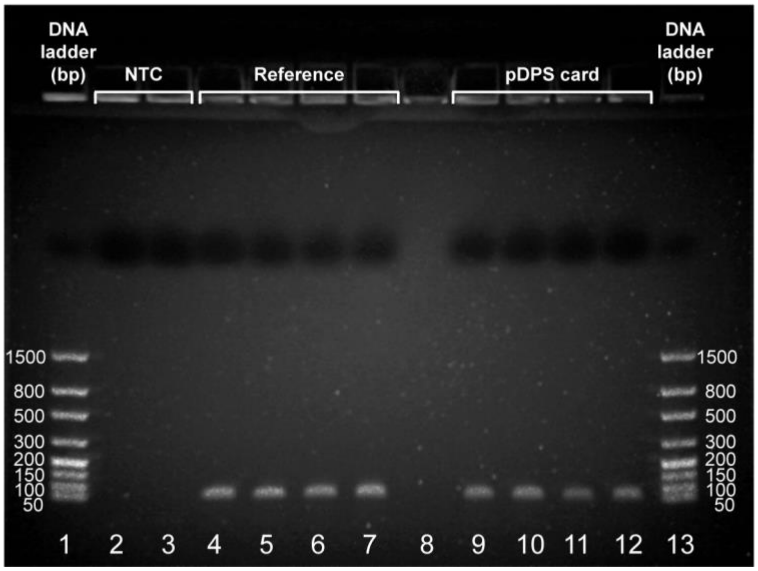

DNA ladder (bp)
NTC
Reference
pDPS card
DNA ladder (bp)
1500
800
500
300
200
150
100
50
1 2 3 4 5 6 7 8 9 10 11 12 13

Table S1. Primers used in the RT-qPCR.

Primer name	Sequence	Product length (bp)
18S Forward	5'-TGTGCCGCTAGAGGTGAAATT-3'	63
18S Reverse	5'-TGGCAAATGCTTTCGCTTT-3'	

References

[1] Schneider, C. A.; Rasband, W. S.; Eliceiri, K. W. NIH image to imageJ: 25 years of image analysis. *Nat. Methods,* **2012**, *9*, 671–675.

[2] Li, X.; Liu, X. Fabrication of three-dimensional microfluidic channels in a single layer of cellulose paper. *Microfluid. Nanofluid.,* **2014**, *16*, 819–827.

[3] Drabkin's reagent product information, *Sigma-Aldrich*, **2015**, (accessed May, 2021).

[4] Armbruster, D. A.; Pry, T. Limit of blank, limit of detection and limit of quantitation. *Clin. Biochem. Rev.,* **2008**, *29*, S49–S52.

[5] DNA purification from dried blood spots (QIAamp DNA mini kit). In *QIAamp® DNA Mini and Blood Mini Handbook*. 2nd edition, 2007; pp 43.
